# Early life shock and labour market outcomes: Panel data evidence from South Africa

**DOI:** 10.1016/j.heliyon.2024.e33529

**Published:** 2024-06-24

**Authors:** Gidisa Lachisa Tato, Assefa Admassie

**Affiliations:** Department of Economics, Addis Ababa University, Addis Ababa, Ethiopia

**Keywords:** Early life shock, Death, Earnings, Unemployment, South Africa

## Abstract

Adverse life events have short- and long-term effects on the livelihood of victims. This paper studies the effect of early life idiosyncratic shocks on labour market outcomes using five rounds of panel data from the National Income Dynamics Study (NIDS) of South Africa. Regression results from alternative panel data estimators suggest that the loss of biological parents early in life is negatively associated with the likelihood of employment and wage earnings. The association is stronger when one loses one's biological mother than one's biological father. Heterogeneity analysis reveals that the loss of a biological father among Black South Africans leads to higher wage earnings compared to other race groups who have experienced the same shock. Education level, perceived health, cognitive ability, and occupation type are strongly associated with the loss of a biological mother, while only education is associated with the loss of a father. These could be the main channels that mediate the link between early life loss of biological parents and labour market outcomes. Therefore, strengthening and aligning child support programmes to reach the victims are required.

## 1 Introduction

Adverse events in life are common in developing countries where their short- and long-term impact on the livelihood of people is paramount. The prominent shocks experienced by households in developing countries include weather shocks [1,2], agricultural commodity price volatility [3], famine [4,5], and armed conflict [6]. Health-related idiosyncratic shocks such as illness and mortality are also worth mentioning [1,7]. Evidence shows that the livelihood systems of victims affected by these shocks are eroded keeping them in perpetual poverty with possible inter-generational impact [[8], [9], [10], [11]]. One of the main reasons why such shocks have a long-lasting impact in a developing country context is due to the lack or shortage of formal insurance mechanisms against such shocks [[12], [13], [14], [15]].

The association between early-life shock and late-life outcomes is a recent phenomenon that has received little investigation. Early life rainfall shock has been identified as a prominent factor affecting health, education, and other socioeconomic outcomes [1,2,16]. Adhvaryu et al. [3] showed the adverse effect of early-life cocoa price volatility on psychological well-being in Ghana. Singhal [6] has also found an adverse effect of early life war experience on mental health. The effect of early life famine experience is also proved to have an impact on the physical height of victims [5], adult health [17], cognitive ability [4], and survival at older ages [18]. Similarly, idiosyncratic shocks such as the loss of parents during childhood are associated with late-life psychiatric disorders [[19], [20], [21]], health, and education [22]. Contemporaneous shocks such as parental unemployment [9] and health shock [10,11] also determine children's education outcomes. Among the very few studies that link idiosyncratic shock with labour market indicators, Börner et al. [23] considered household labour reallocation, while Heltberg and Lund [24] used child labour as an outcome variable.

Though the broader literature suggests the adverse effects of idiosyncratic shocks on the livelihood of victims, it is not obvious if such shocks necessarily lead to long-lasting impacts as there could be insurance mechanisms for mitigation. With very limited formal insurance systems in developing countries, informal insurances are available to mitigate the effect of such shocks [13,[24], [25], [26]]. It is, therefore, worth extending the literature to explore the effect of idiosyncratic shocks on socio-economic outcomes. This paper uniquely investigates the effect of an early-life idiosyncratic shock (loss of biological parents) on late-life labour market outcomes. In doing so, it contributes to the body of literature in development economics by providing empirical evidence on why some people succeed in the labour market while others do not. This is more relevant in a developing country context where labour, which is the main asset for the poor, is the main source of income for the livelihood of individuals and households. For instance, labour income accounts for more than half of the total household income in low- and middle-income countries [27,28]. Fields [27] thus underlines the importance of understanding labour markets and labour earnings in developing countries to understand global poverty. The paper also contributes to the broader shock literature by capturing a permanent shock of parental death unlike most temporary shocks covered by the literature.

This study also contributes to the literature on early-life shock and labour market outcomes in developing countries by utilising very rich panel data from South Africa. South Africa is a great setting for investigating the relationship between orphanhood and late-life labour market challenges, as both are pervasive. South Africa, like other sub-Saharan African countries, has seen a significant increase in orphanages, putting millions of children at risk, particularly as the HIV/AIDS pandemic has spread [[29], [30], [31], [32]]. South Africa, whose formal sector employment accounts for around 70 % of the total employed labour force [33], is also known for its persistently high unemployment rate and wage inequalities. As a result, the formal labour market in South Africa is thought to be the most important predictor of the country's socio-economic status [34]. According to Mosomi and Wittenberg [35], South Africa's aggregate unemployment rate was 31.5 % in 2003, 21.5 % in 2008, and 27.9 % in 2017. The 2020 unemployment rate was 29.4 % [36]. On the other hand, there is a considerable level of wage inequality in the country, with employees in the 90th percentile earning four and six times more than those at the median in 2000 and 2015, respectively [35]. In addition to the availability of rich data, using South Africa as a case study provides an excellent example in terms of capturing the labour market outcomes and drawing relevant lessons for other developing nations.

This paper explores the effect of an early life shock (loss of biological parents in childhood) on wage earnings and unemployment during adulthood using the South African National Income Dynamics Study (NIDS) survey conducted in five rounds (2008–2017). To account for the time-invariant nature of the interest variable, early life loss of biological parents, while accounting for the fixed effect of time-varying covariates, we used the Correlated Random Effect (CRE) estimator proposed by Mundlak (1978) and Chamberlain (1982). The results from the CRE and other alternative estimations reveal the presence of a statistically significant association between the death of biological parents and monthly wage earnings. Adult respondents who have lost their biological mother during early childhood have a 22 % lower monthly wage than their counterparts, while the difference is only about 13 % for those who have lost their biological father. Unemployment is also found to be correlated with the shock variables.

Results from heterogeneous analysis reveal that Black South Africans who lost their biological father early in life earn higher wages than those in other race categories who have experienced the same shock. Alternative mechanisms were also checked to consider how shocks in the early life of the respondents affect their labour market outcomes later in life. We show that the main channel that links the loss of biological parents during early childhood with wage earning is the level of education. However, the perceived health, cognitive ability, and occupation type of respondents are also found to be channels that could explain the link when one loses a biological mother. It is, therefore, important to put safety net programmes in place with financial and psychological support to protect children at the time of death of their biological parents.

The rest of the paper is organized as follows. Section 2 reports the conceptual framework. Section 3 discusses the data and provides basic descriptions of the main variables used in the study. The empirical strategy is presented in Section 4. Section 5 presents and discusses the results from alternative estimators including heterogeneity analysis, robustness checks, and mechanisms. Conclusions and policy implications are presented in Section 6.

## 2 Conceptual framework

In the conventional neoclassical explanation of wage determination market demand and supply of labour play an important role. In this circumstance, labourers decide how much labour to supply based on labour vs leisure hour optimization. However, firms' decision on how much labour to hire depends on factor productivity. That is, individual workers are usually argued to receive compensation equivalent to their productive contribution to a firm's output. Workers who are more productive and therefore contribute more to output would earn higher wages than those with lower levels of productivity. In light of claims that productivity difference is one of the main sources of wage differential among workers, it is natural to ask why there is variation in labour productivity. This leads to human capital theory, which is a standard theory of earnings [37].

Human capital theory, developed by Becker (1964) and Mincer (1974), explains that the wage differential across time and individuals is mainly due to change or variation in workers’ accumulated skills [38]. According to this theory, younger people earlier in their labour market participation earn less due to their engagement in human capital investment which involves an opportunity cost. The stock of human capital accumulated in life, however, is not easy to aggregate from in-school and post-school investments alone as there are other disturbances and initial condition differences that contribute to human capital. Mincer [39] indicates that the initial capacities of individuals and investments provided by the home environment could be the reasons behind the difficulty of aggregation. In support of this, Kao et al. [40] argue that human capital is acquired not only from formal schooling and post-school training but also from family care in preschool years, health, and job search.

Earnings function based on human capital theory broadly suggests that investment in human capital determines wage earnings. More specifically, earning is modelled as a function of years of schooling and age. Years of schooling is a good indicator to capture the first phase of investment in human capital. On the other hand, age is assumed to capture net self-human capital investment activities after completion of schooling [39]. Most previous studies based on this theory consider the measurable components of human capital in explaining wage differentials. For instance, some studies link labour market outcomes to differences in education [[41], [42], [43], [44], [45]], and health situations and shocks [42,[46], [47], [48], [49]].

It is important to note, that the hypothesized association between human capital indicators and earnings hold if individual differences in ability and opportunity, which determines their investment behaviour, persist over their life cycle [39]. This implies that shocks that affect investment in human capital will also affect labour market outcomes. Beyond the effect of shocks during and post schooling, shocks and family situations before schooling are also worth looking at as they affect the early mental and physical development of children. In this regard, the role of parents is considered to be substantial. The transmission mechanism from parent to children could range from the mother's prenatal situations, breastfeeding, and diet to the way children are treated at home. One of the pioneers in human capital theory, Becker (1964), clearly explains the influence of families on the knowledge, skills, values, and habits of their children. According to Becker, a very small difference among children in the preparation provided by their families will lead to growing differences over time [50]. Hence, it is possible to expect significant differences in treatment and preparation between children who lost either or both of their biological parents and those who did not. Such differences are expected to affect human capital development and thereby labour market outcomes. This paper, therefore, adds to the literature by exploring the effect of early-life idiosyncratic shock (loss of biological parents) on late-life labour market outcomes using a very rich data set from South Africa.

## 3 Empirical strategy

We estimate labour market outcomes following human capital theory. In Mincer [39] traditional human capital earnings equation, the wage of an individual can be modelled as a function of years of schooling and other individual characteristics. This earning equation can be specified as [51]:

(1) $$\log\;W_{it}=\alpha +X_{it}\gamma +\mu_{t}+\epsilon_{it}$$

where $\log\;W_{it}$ is the logarithm of wage earnings of individual i in period t. $X_{it}$ is years of schooling and other individual attributes such as age, gender, and race of individual i at time t. $\mu_{t}$ is a time-specific fixed effect and $\epsilon_{it}$ is the composite error term containing individual specific effects and idiosyncratic errors.

In the traditional earnings equation, equation (1), years of schooling is one of the main factors behind variation in earnings. However, in this study, the early life idiosyncratic shock variables are hypothesized to affect labour market outcomes through their effect on human capital formation. Education, the main human capital indicator, correlates with the main variable of interest (early life shock) and outcome variable so including education would result in bad controls. Therefore, to have a clear interpretation of the effect of the shock variables on labour market outcomes, the estimation model is respecified and given in equation (2) as:

(2) $$\log\;W_{it}=\alpha +S_{i}\beta +X_{it}\gamma +\mu_{t}+\epsilon_{it}$$

where $S_{i}$ is a vector of shock dummies measuring whether an individual has experienced early life shock or not. The shock variables considered are the death of the biological father and the death of the biological mother before the age of 5 years. $X_{it}$ now includes both individual and household-specific attributes such as gender, marital status, age, race, and district municipality dummies, but not human capital indicators.

Given the panel nature of the data, random effect and fixed effect estimation approaches are appropriate. The distinction between random effect and fixed effect lies in the randomness of household-specific effects, which might not be the case most of the time. The standard fixed effects model could be used to account for the fixed effect of time-varying covariates. However, in this particular study, due to the time-invariant nature of the main independent variables, running a fixed effects model eliminates the coefficient of time-invariant independent variables. To combat this, as proposed by Mundlak (1978) and relaxed by Chamberlain (1980, 1982), a Correlated Random Effect (CRE) approach helps to account for the fixed effects of time-varying covariates while keeping the estimates of time-constant variables. More specifically, the approach has the flexibility advantage to estimate the effect of time-varying variables while providing effect estimates of time-invariant variables that are unbiased due to a possible correlation with time-varying unobserved heterogeneity [52,53]. In the setup of this study, where panel data is used with time-invariant main independent variable, the effect of early life shock exploits the cross-sectional variation once the time and cross-sectional variations attributed by other time-variant and time-invariant control variables are accounted for. Therefore, the CRE model which is the main estimation approach for this study is specified as:

(3) $$\log\;W_{it}=\alpha +S_{i}\beta +Z_{i}\theta +X_{it}\gamma +{\bar{X}}_{i}\delta +T_{t}\mu +\varepsilon_{it}$$

where $S_{i}$ is a vector of shock dummies which are time-invariant. $Z_{i}$ is a vector of other time-constant factors like gender and race. $X_{it}$ includes independent variables which change both across individual i and time t. The new term ${\bar{X}}_{i}$ is the vector of time averages of time-varying variables. $T_{t}$ is a vector of aggregate time effects and $\varepsilon_{it}$ is the error term.

Identification in both the fixed and random effects estimators requires the strict exogeneity assumption. In addition, the random effects estimator requires the orthogonality assumption. Therefore, the specification in equation (3) estimated using the CRE model that accounts for the fixed effect of time-varying regressors requires at least the shock variables to be exogenous, which is still hard to make. Therefore, the paper establishes an association between early-life shock and late-life labour market outcomes. However, causal implications are also drawn based on alternative estimations and hypothesis validations.

## 4 Data

This study uses five waves (2008–2017) of data from the National Income Dynamics Study (NIDS) of South Africa. In the first round in 2008, data was collected from more than 28,000 individuals in about 7000 households across the country. The subsequent waves (2010/11, 2012, 2014/15 and 2017) used this initial sample of household members as Continuing Sample Members (CSM). Children born to CSM mothers are added as CSMs and tracked in subsequent waves. Other household members in the subsequent waves are considered Temporary Sample Members (TSMs) and not tracked. This study considers adult respondents from the continuing sample observed in at least two of the NIDS waves. 73 % of the individuals from wave 1 were successfully interviewed in wave 5.77 %, 87 %, and 92 % of the individuals added as CSMs in the subsequent three waves were successfully interviewed in wave 5, respectively [54]. For this study, only adult respondents in the working-age group (15–64 years inclusive) are selected. To utilize the panel nature of the data, 16,923 uniquely identified working-age respondents observed in at least two waves and with full information, which sum to a total of 62,915 observations, are used for the analysis.

Our main dependent variable is wage earnings, though an unemployment dummy is also used as an alternative indicator. Total monthly take-home pay (earnings) is derived from primary and secondary employment, casual work, and self-employment through aggregation. Early life loss of biological parents, which is an idiosyncratic shock, is the main variable of interest hypothesized to affect labour market outcomes. The death of a biological father and mother before the respondents celebrated their fifth birthday are taken as regressors separately. A cut-off point of 5 years can be justified for two main reasons. First, at the time the shocks occurred the children were not eligible for schooling. In South Africa, education is compulsory for children between 5 and 15 years of age, with an official minimum age of 5 years old for starting preparatory grade (Grade R/0) [55]. Second, events before the age of five years have been shown to have a permanent effect on adult outcomes, particularly in shaping human capital development [56]. As a robustness check, the age of 15 years (before respondents are formally eligible to enter the labour market) is also used as a cut-off point to construct the idiosyncratic shocks.

In the estimations, other control variables such as age, gender, marital status, race, province/district municipality, and time dummies are included to account for their effect. Though the focus of this paper is to thoroughly analyze the association between parental death and labour market outcomes, controlling for the attributes of other potential factors is important. For instance, the pioneer Mincer's [39] traditional human capital equation considers age, gender, and race as potential attributes to earnings [51]. Marital status is also considered an important factor behind variation in labour market outcomes [40,43,44,47]. Province and time dummies are included to account for region- and time-specific effects on labour market outcomes, respectively. Education level, perceived health, emotional well-being disorder, occupation type, and cognitive ability are used as possible mechanisms linking shocks with labour market outcomes. That is, early-life parental death, through its effect on human capital formation and related outcomes, could affect adulthood labour market outcomes.

## 5 Results and discussion

### 5.1 Descriptive statistics

Table 1 presents summary statistics of key variables for the pooled data set including the between and within standard deviations. With the notion of weather shocks and deaths of family members are commonly occurring shocks in developing countries, and in South Africa, as indicated in Column (1), about 18 % of the respondents lost their biological father before they celebrated their 15th birthday, while about 8 % lost their biological mother by the same age. Loss of a father and mother in early childhood (before the age of 5 years), on the other hand, is limited to about 6 % and 2 %, respectively.

**Table 1:** Summary statistics of pooled data.

	(1)	(2)	(3)	(4)
	Mean	sd (Between)	sd (Within)	Obs.
*Main Variables*				
Father deceased before age 5	0.06	0.229	0	62915
Mother deceased before age 5	0.02	0.134	0	62915
Father deceased before age 15	0.18	0.388	0	62604
Mother deceased before age 15	0.08	0.271	0	62812
Monthly earnings (in SA Rand)	4043.90	5552.58	5075.07	24472
Unemployed dummy	0.16	0.219	0.230	62915
*Other Controls*				
Age	34.11	14.036	2.895	62915
Female	0.58	0.497	0	62915
Married	0.31	0.410	0.207	62915
Widowed or separated	0.07	0.218	0.132	62915
Single	0.61	0.444	0.193	62915
African	0.82	0.394	0	62915
Coloured	0.14	0.355	0	62915
Asian/Indian	0.01	0.114	0	62915
White	0.03	0.174	0	62915
*Mechanisms*				
Education level	9.97	3.670	0.845	62782
Perceived health	3.83	0.741	0.799	62826
Emotional well-being disorder	6.95	2.723	3.536	61611
Elementary occupation	0.33	0.424	0.242	23705
Financial literacy	2.14	1.078	0	12645

*Notes:* Column (1) reports the mean values of key variables. Columns (2) and (3) are the between and within standard deviation of the panel data, while column (4) shows the total number of observations for each variable. Data on financial literacy is available only in the last (2017) wave of the NIDS survey.

The higher rate of death of men over women is consistent with data from the official South African statistical reports. According to a report by Maluleke [57], for the period between 1997 and 2016 death of males was consistently higher than the death of females. For instance, the difference in the proportion of death between males and females for the years 1997–2000 was on average about 8.8 percentage points. During the same period, the main cause of death (more than 50 % of deaths on average) in the country was due to non-communicable diseases, with a smaller proportion of death resulting from covariate shocks falling into the categories of war, famine, and other natural disasters. This fact reveals the importance of considering death due to idiosyncratic shocks and evaluating its long-term consequences.

The pooled data indicates that about 16 % of the sample of respondents are unemployed, while the remaining are either economically inactive (43 %) or employed (41 %). The average monthly earning of those who are employed, as presented in Column (2), is about 4044 South African Rand (this is about 494 USD based on an average official exchange rate of 8.19 Rand per USD in the panel survey year of 2012). There is more than one-fold variation in earnings both between and within individuals over time. There is consistently high growth in the annual earnings over time (see Fig. 2), with average monthly earnings in the most recent wave outweighing the base year by about 83 %. There is also a very clear and growing difference in the average monthly earnings by education categories (see Appendix Table A1). The variation in earnings within each education group is larger than the average value by more than one-fold. In addition, the variation in wage earnings increases by education level.

**Fig. 1:**
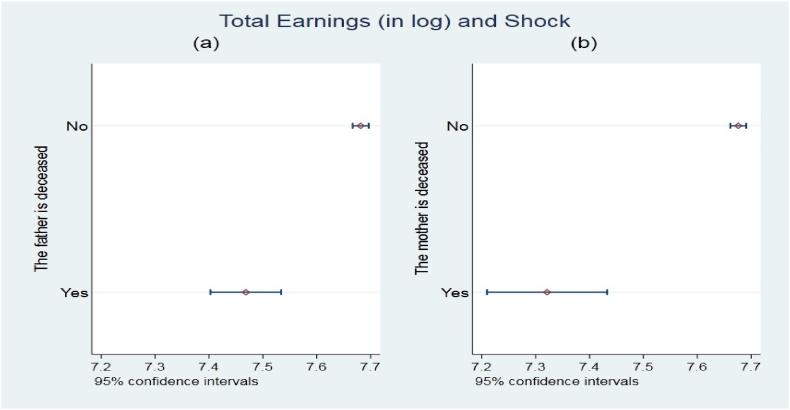
Earnings differences by shock types. *Notes:* Panel (a) relates the average monthly earnings and loss of a biological father, while Panel (b) considers the loss of a biological mother.

**Fig. 2:**
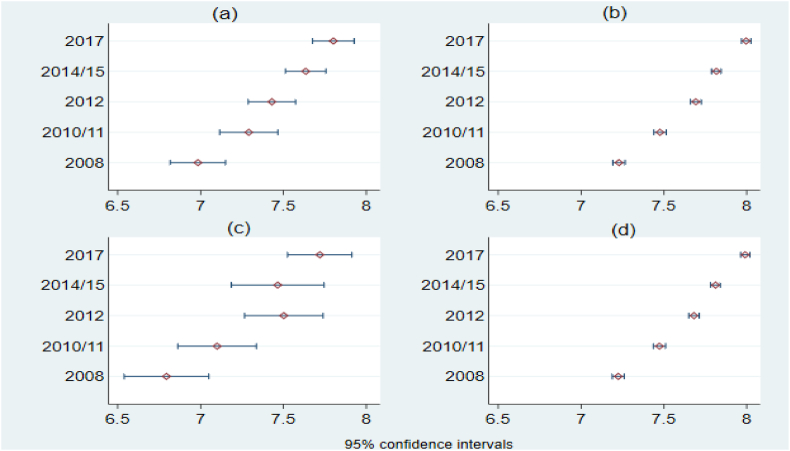
Earnings variation (in log) by waves and shock types. *Notes:* The figure shows trends in average monthly earnings (in log) across the waves. Panels (a) and (c) are the trends for the groups who have lost their father and their mother in childhood, respectively. Panels (b) and (d) are the trends for those who have not lost their parents.

The demographic information from the aggregate data, presented in Table 1 also shows that the average age of the respondents is about 34 years with the majority of them being female (58 %), single (61 %), and African (82 %). Among the variables considered to capture the mechanisms through which early life shock might contribute to labour market outcomes later in life, average years of schooling is about 10 years; perceived health evaluated in a 1 to 5 scale is on average 3.83 and emotional well-being disorder based on the Center for Epidemiologic Studies Depression Scale (CES-D 10) is 6.95. About 33 % of the respondents have elementary occupations, while the average financial literacy score is 2.14 out of 5.

Fig. 1(a and b) presents the difference in total monthly earnings by shock experience. As shown in Fig. 1(a) and (b), there is a statistically significant difference in the mean earnings between those who have lost their biological father and mother early in their lives and those who did not, respectively. In both cases of loss of biological father and mother, the average monthly wage earnings are lower among those who have experienced the shock. The age cohort analysis (see Appendix Table A2) reveals an interesting result where the average monthly wage-earning difference between the two groups increases statistically and economically with age. It is, therefore, imperative to expect an association between the loss of biological parents and labour market outcomes.

Variation in monthly total earnings by wave and shock type is also presented in Fig. 2(a–d). The figure depicts first, the average earnings of those who have lost either their father or mother (as presented in Fig. 2(a) and (c), respectively) is lower in each wave compared to the control groups (presented in Fig. 2(b) and (d)). Second, there is no statistically significant difference in the earnings of those who have lost their parents across the consecutive waves, while there is a statistically significant difference in earnings growth between those who have not experienced the shocks. With this background information, we now try to explore the effect of the shock variables on the stated labour market outcomes.

### 5.2 Main findings

Table 2 presents alternative estimation results of the effect of the loss of biological parents in childhood on labour market outcomes later in life. All the estimations include other control variables such as age, age squared, gender, marital status, race, and province. In these estimations cluster standard error using district municipality is used. Column (1) reports estimations results using a pooled cross-sectional ordinary least square model. The estimation result reveals that both the death of the biological father and the death of the biological mother in early life have a statistically significant association with the total monthly earnings of the respondents during adulthood. To utilize the panel structure of the data, with time-invariant regressors of the variables of interest, a random effects model is estimated and presented in Column (2). Like the first estimation, both shocks have statistically significant associations with wage earnings. The random effects model has weakness in terms of controlling time-invariant unobserved heterogeneities, while the fixed effect solves the problem at the expense of dropping the coefficients of time-invariant regressors. As a result, a CRE model proposed by Mundlak (1978) and Chamberlain (1980, 1982) is used to account for the fixed effect of time-varying covariates while keeping the estimates of time-constant variables.

**Table 2:** The effect of early life parental death on earnings and unemployment.

	(1)	(2)	(3)	(4)	(5)
	POLSEar	REEar	CREEar	HeckEar	ProbUnem
*Panel A - Earnings (in log)*					*Unemp.*
Death of a Father	−0.136∗∗	−0.155∗∗	−0.133∗∗	−0.153∗∗∗	0.072∗
	(0.050)	(0.048)	(0.044)	(0.046)	(0.034)
Death of a Mother	−0.206∗∗	−0.216∗∗∗	−0.219∗∗	−0.221∗∗∗	−0.151∗
	(0.075)	(0.064)	(0.073)	(0.058)	(0.061)
*Panel B - Employed Dummy*					
Death of a Father				−0.229∗∗∗	
				(0.053)	
Death of a Mother				−0.414∗∗∗	
				(0.078)	
Time Fixed Effects	Yes	Yes	Yes	Yes	Yes
Municipality Fixed Effects	Yes	Yes	Yes	Yes	Yes
Other Controls	Yes	Yes	Yes	Yes	Yes
Observations	24472	24472	24472	62915	62915

*Notes:* Alternative earning estimation results are provided in Columns (1) to (4), while a Panel Probit estimation for the unemployed dummy is given in Column (5). Column (1) and Column (2) report estimation results from pooled ordinary least square and random effect models, respectively. In these estimations, cluster standard error using district municipality is used. Column (3) presents the estimation of the main model, a Correlated Random Effect model. A Heckman selection model is depicted in Column (4), where the result from the Probit estimation of the Heckman model is given in Panel B. Control variables used in the estimations, other than indicated, include age, age squared, gender, marital status, and race. For estimations in Columns (3) to (5) robust standard errors are used once the district municipality replaces the province as control.

∗ p < 0.05.

∗∗ p < 0.01.

∗∗∗ p < 0.001.

Column (3) presents the estimation results from the CRE model, where the deaths of the biological father and mother are statistically significant in explaining wage earning variations. The earnings figure of those who have lost their father is lower by about 13.3 % than for those who have not experienced the shock. Consistently, the earnings amount of those who have lost their biological mother during childhood is lower on average by about 22 % than for those who have not lost their mother. Previous studies that explored the effect of early life shock on late life economic outcomes show consistent results, but with a lower magnitude. For instance, Isen et al. [58] show a 0.1 % reduction in adult annual earnings due to an extra day with mean temperatures above 32°C in utero, while Tien and Adoho [59] found a 2.5–3.5 % difference in overall inequality of adulthood earnings due to early adulthood (ages 18–25) exposure to intensely violent conflict. According to Beegle et al. [22], maternal orphanhood leads to 8.5 % lower consumption expenditure during adulthood. In this and prior estimations, the impact of the death of a mother in early life is higher in magnitude than that of the death of a father. This could be due to the greater role mothers play in the human capital formation of their children.

Gender roles and differences have always been a point of discussion with varying evidences across countries. Though there is progress in gender equality as labour force participation of women and mothers is growing [60,61], the contribution of women in housework and childcare is still greater than the contribution of men, particularly in developing countries [62,63]. Such variation arises due to differences in individual-level variables such as relative resources, time availability, and gender ideology. In addition, macro factors such as economic development, female labour-force participation, gender norms, welfare regimes [64], and cultural difference [61] among others, explain gender gaps. On top of the greater responsibility that women bear concerning housework and childcare, resources controlled by women are mostly geared towards increased household expenditures on inputs into child well-being, including nutrition, education, and health services [[65], [66], [67], [68]]. The evidence that the loss of a mother harms human capital and thereby labour market outcomes in the later life of children is a clear indication of the contribution of women in the human capital development of their children.

In our earnings equation, we consider respondents who receive wage earnings from employment (including self-employment). However, the experience of shocks in early childhood that affect human capital might deter working-age respondents from labour market participation. If that is the case, the estimations run earlier may suffer from the problem of sample selection. To account for this, Heckman [69] suggests a two-step estimation. Accordingly, the first model estimates the probability of participation in the labour market using Probit regression, while the second model estimates the earning equation, considering the result of the former estimates. Identification in the Heckman model requires a valid exclusion restriction in the selection equation, where the excluded variable needs to affect selection but not the outcome. Different studies used different variables to satisfy the exclusion restriction criteria. In this study, household size was included in the selection equation but excluded from the earning equation. The justification for the use of this variable is that it might affect the likelihood of employment participation, but an individual's earning level is unlikely to depend on family size. In addition, different studies used the number or presence of children, which is highly correlated with family size, in the selection equation to satisfy the exclusion restriction [[70], [71], [72]]. Considering the lack of tests for the validity of the instrument used and possible concerns about the justification, the study is limited to describing associations without making causal inferences.

Column (4) of Table 2 presents the estimation results based on Heckman selection approach using household size as an additional control in the selection equation. The results are consistent with all the above alternative estimations. In the Probit estimation of the Heckman model, the death of a father and the death of a mother both have a statistically significant adverse effect on the probability of working. Those who have lost their biological mother in childhood have about 41 % lower probability of being employed than their counterparts. Similarly, the difference is about 23 % in the case of the death of a biological father.

We also estimate the probability of being unemployed as a function of the shock variables and other controls using a panel Probit model and report the results in Column (5) of the same table. The results suggest that those who lost their father have a higher probability of being unemployed, while those who lost their mother have a lower probability compared to their respective counterparts. Combining this result with the Probit estimation from the Heckman estimator shows that those who have lost their mother have a lower probability of being both employed and unemployed. This implies that the loss of a mother seems to put the majority in the economically inactive category compared to their counterparts. This could be due to the lower likelihood of those who have lost their mother being in a good position in terms of their human capital status [22]. However, for those who have lost their father, while the probability of being employed is lower, the likelihood of being unemployed is higher. This means they are striving to find a job but with a lower probability of obtaining employment. This could be due to the two-sided effect of the loss of a biological father in early life. First, higher tendency for the household to experience income shock following the loss of a father, as in most cases the relative household income share of husbands is higher [73]. This is more likely true (with a higher share) in developing countries, particularly in a society where males are considered the main breadwinner. Second, children have a higher chance of human capital development if the surviving parent is a mother as a mother's contribution to parenting is usually greater. In these contradictory situations, children with some human capital are forced to enter the labour market earlier to support the household economically though they have a lower tendency to obtain employment.

In the discussions so far, the effects of the death of a biological father and the death of a biological mother were analysed independently to see how they relate to later earnings. Nevertheless, the interaction term between the death of a father and the death of a mother which captures the effect of the loss of both biological parents is found to be statistically insignificant. This could be due to a sample size problem as the proportion of those who have lost both of their biological parents in early childhood accounts for less than 0.5 % of the total observation. The other reason could be due to interventions, like foster child grants or orphanhood projects in South Africa, which are intended to protect only children who have lost both of their biological parents in their childhood.

It is also worth investigating whether the effect of such shocks varies across socio-demographic factors. In line with this, heterogeneity analyses using gender and race are estimated and presented in Table 3. Columns (1) and (2) report the interaction between the shock variables and gender to check if there is variations in the effect of the shocks on wage earnings between male and female respondents. As discussed earlier, for multifaceted reasons women bear more burden at home than men, particularly in developing countries, and we, therefore, expect the effect of the shock to varying by gender. The heterogeneity analysis using a Correlated Random Effect model reveals no statistically significant difference in the effect of the shock by gender. Only at the margin (10 % level of significance) do women who have lost their mothers in childhood earn lower wages than men who have lost their mothers in childhood.

**Table 3:** Early life Parental Death and Earnings - Heterogeneity Analysis.

	(1)	(2)	(3)	(4)
	FathGender	MothGender	FathRace	MothRace
Death of a Father∗Female	−0.096			
	(0.087)			
Death of a Mother∗Female	−0.277†			
		(0.150)		
Death of a Father∗African		0.297∗		
			(0.123)	
Death of a Mother∗African				0.153
				(0.157)
Death of a Father	−0.079	−0.132∗∗	−0.376∗∗∗	−0.133∗∗
	(0.060)	(0.044)	(0.114)	(0.044)
Death of a Mother	−0.216∗∗	−0.039	−0.215∗∗	−0.334∗
	(0.073)	(0.120)	(0.073)	(0.131)
Female	−0.361∗∗∗	−0.362∗∗∗	−0.366∗∗∗	−0.366∗∗∗
	(0.019)	(0.019)	(0.019)	(0.019)
African	−0.543∗∗∗	−0.543∗∗∗	−0.554∗∗∗	−0.545∗∗∗
	(0.035)	(0.035)	(0.035)	(0.035)
Time Fixed Effects	Yes	Yes	Yes	Yes
Municipality Fixed Effects	Yes	Yes	Yes	Yes
Other Controls	Yes	Yes	Yes	Yes
Observations	24472	24472	24472	24472

*Notes:* Column (1) and Column (2) report CRE estimation results once the interaction term between the death of a father and the death of a mother is incorporated with the gender of the respondent (female dummy). In the same way, Column (3) and Column (4) introduce the interaction term of the respective shock variables with the race of the respondents (black South African dummy). Other control variables used in the estimations are age, age squared, gender, marital status, and race. Robust standard errors are given in parentheses.

† p < 0.10.

∗ p < 0.05.

∗∗ p < 0.01.

∗∗∗ p < 0.001.

Racial discrimination is an important legacy of apartheid in South Africa which was in place until 1994. Though anti-discrimination legislation has been in place since then, racism and its manifestations remain an issue in the country [74,75]. Discriminatory actions due to racism are expected to have psychological, social, political, and economic consequences. For instance, Oliver et al. [76] indicate a clear difference in earnings and wealth between black and white South Africans. The results from the main estimation in this study are consistent with the literature, where the earnings of the majority of Black South Africans are lower on average by about 54 % than the other race categories. Here it is also interesting to see the existence of a difference in the effect of the shocks between race categories, with the assumption that Black South Africans are discriminated against social and economic insurances (formal or informal) following the loss of their biological parents. The interaction term between the death of a biological father and a black South African dummy reveals a result contrary to our expectation. Black South Africans who lost their father during childhood earn higher wages than those in other race categories who have experienced the same shock. On average, Black South Africans who have lost their father earn a wage higher by about 30 % than the other race groups who have lost their biological father too. Though thorough and further investigations are required, one of the reasons could be the opportunity provided by the Expanded Public Works Programme (EPWP) and other government job creation programmes in South Africa. The EPWP, for instance, aims to provide short-to medium-term employment opportunities and income transfers to poor households. With this in mind, about 90 % of the beneficiaries of the South African EPWP and other government job creation programmes are black South Africans [36]. Therefore, this might have provided a better opportunity for those black South Africans who have experienced paternal death and are economically deprived than the other race groups who have experienced the same shock.

### 5.3 Robustness checks

The previous estimations considered shocks experienced by respondents before their fifth birthday. The results presented in Column (1) of Table 4 are from the shock experienced before the respondents were formally eligible to participate in the labour market, to check for consistency of the results. For this, whether respondents lost their biological mother and father before age 15 is used as the main independent variables. As the estimation result reveals, both the death of a biological father and mother are statistically significant in explaining wage differences, though the magnitude of the effect is lower than if the shock is experienced before the age of 5. The death of a biological father results in an average wage that is lower by about 7 %, while the death of a mother results in lower wage earnings of about 16 %. In general, consistent with the results of the main estimation, the loss of biological parents has a detrimental effect on labour market outcomes, where the effect of the loss of a biological mother is higher than the effect of the loss of a biological father.

**Table 4:** Robustness checks.

	(1)	(2)	(3)	(4)
	DBef15	CovShock	Attrit	HTaylor
Death of a Father	−0.070∗∗	−0.131∗∗	−0.133∗∗	−4.760∗∗
	(0.025)	(0.044)	(0.044)	(1.507)
Death of a Mother	−0.164∗∗∗	−0.222∗∗	−0.219∗∗	−8.325∗∗
	(0.040)	(0.073)	(0.073)	(2.944)
Wave freq.			0.009	
			(0.041)	
Time Fixed Effects	Yes	Yes	Yes	Yes
Municipality Fixed Effects	Yes	Yes	Yes	Yes
Other Controls	Yes	Yes	Yes	Yes
Observations	24338	24472	24472	24472

*Notes:* Column (1) presents CRE estimation of the effect of the death of a biological father and the death of a biological mother before the age of 15 on wage earnings. Column (2) reports CRE estimation results once the interaction term between the place of birth and year of birth is introduced as an additional explanatory variable. This helps to disentangle idiosyncratic shocks from covariate shocks. Column (3) shows a test for attrition bias based on the main estimation model. Column (4) presents the Hausman-Taylor estimator. Other control variables used in the estimations are age, age squared, gender, marital status, and race. Robust standard errors are given in parentheses.*p < 0.05.

∗∗ p < 0.01.

∗∗∗ p < 0.001.

There are concerns related to the consistency and validity of the results from the above estimations. One of the main concerns is whether the results are attributed to idiosyncratic shocks, which might be confounded with covariate shocks. To disentangle the effect of idiosyncratic shocks from covariate shocks, an interaction term between respondents’ year of birth and place of birth is introduced as a means to control the effect of covariate shocks, with the assumption that no mobility of parents occurred before the child was 5 years old. These variables are considered good proxies for the year and place of death of the parents, respectively. Column (2) presents estimation results once the interaction term is introduced as an additional control into the Correlated Random Effect model with robust standard error. The result is still consistent with estimations from the main model, where both the death of a biological father and mother are statistically significant in explaining earnings variations. The magnitude of the effect is also similar to the coefficients from the main estimation model.

The second aspect to be checked to confirm the reliability of estimation results in a panel data setup is attrition bias. This study considers unbalanced panel data with observations in at least two waves. As described in Section 4.1, the attrition rate from wave 1 to wave 5 is 27 %. Thus, attrition might bias the estimation results. To check this, Wooldridge [77] suggests the inclusion of lead or lag of the selection (time) variable or the number of subsequent waves observed in the dataset to test their significance. To preserve the number of observations used in the main estimation, the number of waves is introduced as an additional explanatory variable. The estimation result presented in Column (3) of the table reveals that the introduced variable is statistically insignificant. Therefore, the estimation result from the main model is not subjected to attrition bias and is reliable.

The third and most important concern for causal interpretation is the randomness of the shock variables. Acknowledging the non-random nature of the shock variables, alternative estimations and hypotheses are tested to validate the results. The main estimation model in this study, the correlated random effects model, has the advantage of providing effect estimates of time-invariant variables that are unbiased by a possible correlation with time-varying unobserved heterogeneity. However, this is based on a strong assumption that the time-invariant variable is uncorrelated with time-invariant unobserved heterogeneity. That is, the main time-invariant independent variable is assumed exogenous [53]. In this case, an approach proposed by Hausman and Taylor [78] provides an internal instrument to consistently estimate the coefficient of time-invariant endogenous variables, the shock variables in this study. This approach also requires that the instruments should be uncorrelated with individual effects and the idiosyncratic error terms. The estimation result, presented in Column (4), is still consistent in terms of sign and significance, though we have inflated coefficients and standard deviations. This is due to the weak instruments that cause serious size distortions. For instance, the inclusion of parents’ education in the model removes about half of the size distortion.

To check for an alternative hypothesis which could explain the death of biological parents and at the same time earnings are tested and presented in Table 5. Random effects models are run for comparison, with the simple random effect model presented in Column (1). The first question could be “What if the death of biological parents was due to hereditary diseases which transmit to their offspring and make them economically less active?“. If the early age death of parents is due to hereditary diseases, we also expect that to happen to their children or at least would make their children economically inactive earlier than others and exit the labour market. There is some evidence documenting a lower life expectancy for those who suffer from hereditary diseases [79,80]. On the other hand, data from the World Bank indicates that the average life expectancy at birth in South Africa for the period between 1960 and 2005 was about 56.5 years. We, therefore, expect the wage differential to die out with the increasing age categories, particularly for the age category above 45 years. The result presented in Table 5 in Columns (2) to (4), however, is contrary to our expectations statistically and economically. It implies that shocks experienced in early childhood have a persistent and growing effect on labour market outcomes. Contrary to our argument, if the hereditary disease makes the victims less productive while they stay participating in the labour market could lead to a higher wage differential.

**Table 5:** Early life parental death and earnings - Sensitivity analysis.

	(1)	(2)	(3)	(4)	(5)	(6)	(7)	(8)
	Simple	A15-30	A30-45	A45+	Inc	POcc	FBA5	MBA5
D. Father	−0.218∗∗∗	−0.079	−0.186∗∗	−0.342∗∗∗	−0.203∗∗∗	−0.299∗∗∗	−0.143∗	
	(0.053)	(0.091)	(0.060)	(0.094)	(0.051)	(0.061)	(0.067)	
D. Mother	−0.366∗∗∗	−0.296∗∗	−0.233	−0.500∗∗∗	−0.347∗∗∗	−0.294∗∗		−0.234∗∗
	(0.079)	(0.109)	(0.122)	(0.149)	(0.078)	(0.110)		(0.086)
Controls	No	No	No	No	Yes	Yes	No	No
Obs.	24472	7357	9896	7219	23850	12547	3852	1448

*Notes:* Column (1) reports a base model which estimates the effect of idiosyncratic shock variables (death of father and death of mother before age of 5) on monthly wage earnings. Columns (2) to (4) decompose the prior estimation by age category to check if the effect of the shock variables is vanishing with age. Columns (5) and (6) control the effects of the income step of parents at the 15th age of the respondent and occupation category of parents, respectively. Columns (7) and (8) compare the effects of the loss of parents before age of 5 with the loss of parents between the ages of 5 and 15. Due to the time-invariant nature of the independent variables in these estimations random effects model is used with clustered standard errors (given in parentheses).

∗ p < 0.05.

∗∗ p < 0.01.

∗∗∗ p < 0.001.

The second question we ask is “What if the economic status of parents is the reason for their death and could also contribute to the nature of the later wage earnings of their children through human capital investment or bequests?“. If that is the case, we expect introducing the income level of parents into the earnings equation in addition to the shock variables to erode the effect of the latter. To empirically check this, the income of parents at the age the respondents were qualified to enter the labour market is considered due to the lack of data on income at the early age of the respondents and the regression results are presented in Column (5) of Table 5. The result reveals marginal difference in the effect of the shock variables compared with the estimation in Column (1), which invalidates our hypothesis. The other hypothesis is the role of the occupation type of parents in determining the wage earnings of their children. What if the occupation type of the parents is the reason for their death and their children's subsequent earnings, given there is a high possibility that children will follow the occupation type of their parents? Here as well, we expect the effect of the shock variables to drop at least in magnitude once the occupation type of parents is controlled for. The result as presented in Column (6), however, doesn't meet this expectation consistently.

Finally, one of the other options to check for the importance of loss of biological parents early in life in determining late-life labour market outcome is to compare groups who have experienced the shock before the age of 5 and after the age of 5 (but before the age of 15). In this scenario, it is hard to argue that the death of parents before the respondents' 5th birthday has unique characteristics compared to parental death after the child's 5th birthday. A comparative analysis presented in the last two columns of the table shows that the effect of early childhood shock (before age of 5) is statistically significant in explaining variation in wage earnings in comparison to shocks experienced in middle and late childhood (age between 5 and 15 years). The effect of the loss of a mother in early childhood is both economically and statistically higher than the loss of a father. In general, accounting for possible sources of endogeneity to check for consistency of the results, the loss of biological parents early in life has a significant effect on later labour market outcomes.

### 5.4 Mechanisms

There is ample literature supporting a strong association between human capital and labour market outcomes. Positive labour market outcomes are highly likely to occur whenever there is a high investment in human capital early in life. On the other hand, shocks which hamper investment in children are expected to lower their labour market outcomes. Accordingly, in the above analysis, we document the adverse relationship between idiosyncratic shocks and labour market outcomes. The main channel, as described above, is expected to happen through human capital development. Given this, we consider education attainment, perceived health, emotional well-being disorder and cognitive ability as the main possible channels through which shocks affect labour market outcomes. In addition, the occupation type of respondents is also checked as a possible mechanism.

Table 6 reports estimations based on alternative outcome variables only for respondents with positive wage earnings (employed sample). The first three estimations are based on the correlated random effect model with outcome variables of education level (measured in years of schooling), perceived health (measured in the range of 1 (very low) to 5 (very high)), and emotional well-being disorder (measured using the Center for Epidemiologic Studies Depression Scale (CES-D 10)), respectively. Column (4) presents regression results from a panel probit estimator considering an elementary occupation dummy as an outcome variable. Lastly, information on financial literacy (measured in the range of 1 (very low) to 5 (very high)) becomes available in the fifth wave of NIDS data, and OLS estimation is used on this variable, and the results are presented in column (5) of the table. In this estimation, financial literacy is used as a proxy for cognitive ability, as supported by literature that documented the existence of a strong association between financial literacy and cognitive ability [81,82].

**Table 6:** Mechanisms - employed sample.

	(1)	(2)	(3)	(4)	(5)
	Education	PHealth	EmoWellbeing	OccuElemd	FinLitw5
Death of a Father	−0.493∗∗	−0.012	0.039	0.103	−0.049
	(0.154)	(0.033)	(0.127)	(0.099)	(0.064)
Death of a Mother	−1.186∗∗∗	−0.160∗∗	0.290	0.489∗∗	−0.394∗∗∗
	(0.273)	(0.060)	(0.234)	(0.181)	(0.113)
Time Fixed Effects	Yes	Yes	Yes	Yes	No
Municipality Fixed Effects	Yes	Yes	Yes	Yes	Yes
Other Controls	Yes	Yes	Yes	Yes	Yes
Observations	24416	24429	23896	23632	5786

*Notes:* Columns (1) to Column (3) report CRE estimation results with education level, perceived health, and emotional well-being disorder as outcome variables, respectively. Column (4) shows panel Probit estimation using an elementary occupation dummy as a dependent variable. Column (5) presents the OLS estimation of financial literacy as an outcome which is available only in wave 5. Other control variables used in the estimations are age, age squared, gender, marital status, and race. Robust standard errors are given in parentheses.*p < 0.05.

∗∗ p < 0.01.

∗∗∗ p < 0.001.

Column (1) presents education level as one of the main mechanisms to link the loss of parents in early life with labour market outcomes. As the result reveals, education level is negatively associated with the death of both biological father and mother. The education level of those who have lost their father in childhood is lower by about 0.5 than those who have not lost their father. The difference for those who have lost their mother is more than twofold higher than for those who have lost their father. That is, the educational attainment of those who have lost their mother is about 1.2 years lower than those who have not lost their mother. This figure accounts for about 12 % of the average education level of all the respondents in the sample. This implies the contribution of surviving mothers to the education of their children. Consistently, the contribution of mothers in maintaining and improving their children's health is paramount. In support of this, as given in Column (2) of the table, only the death of a mother is statistically significant in explaining differences in perceived health among the respondents. In line with these, cognitive ability, proxied by financial literacy, is explained again only by the death of a mother (see Column 5). However, the death of a father or the death of a mother is found to be statistically insignificant in determining emotional well-being disorder. In this study, emotional well-being disorder is measured by the Center for Epidemiologic Studies Depression Scale (CES-D 10) developed by Radloff [83], which is one of the most commonly used measures of mental health disorder. A consistent result is obtained by considering if the shock (death of parents) happened before the age of 15 rather than before 5 years of age (see Appendix Table A3).

Individuals in the labour force with lower levels of human capital are expected to have less chance of getting employment. If they do secure employment, they have a higher probability of being employed in elementary occupations than as professionals. Therefore, if the death of biological parents adversely affects the human capital development of the victims, they are more likely to be employed in elementary jobs. The result presented in column (4) of the table is partly in support of this hypothesis where those who have lost their mother have a high probability of having an elementary occupation, while the result is insignificant for the death of a father. It is also important to note that the loss of biological parents might completely lock the victims into economically inactive or unemployed groups. It is, therefore. worth checking the validity of the mechanisms for all sample respondents.

All sample respondents are considered in checks for consistency of the mechanisms, as presented in Table 7. The results are robust to what we have presented in Table 6 except that in the current estimation death of a mother is statistically significant in explaining emotional well-being disorder. That is, those who have lost their mother have higher rates of emotional well-being disorder than their counterparts. Overall, the death of a father and the death of a mother are both found to be statistically significant explanation for labour market outcomes, though the magnitude of the effect is higher for the loss of a biological mother in early childhood. Consistently, the death of a mother is statistically and economically significant in explaining variation in the channels, while education level is the only channel among the alternatives that links the loss of a father with wage earnings. Therefore, due attention should be given to those children who have lost their parents, with more emphasis on those who have lost their mother in early life, to minimize the adverse effect on their future livelihoods as it is likely to affect the livelihood of the succeeding generation and the economy of the country [[8], [9], [10], [11]].

**Table 7:** Mechanisms - all sample.

	(1)	(2)	(3)	(4)
	Education	PHealth	Emowellbeing	FinLitw5
Death of a Father	−0.403∗∗∗	−0.032	0.116	−0.025
	(0.109)	(0.021)	(0.080)	(0.042)
Death of a Mother	−0.975∗∗∗	−0.117∗∗∗	0.278∗	−0.245∗∗∗
	(0.192)	(0.035)	(0.122)	(0.071)
Time Fixed Effect	Yes	Yes	Yes	No
Municipality Fixed Effect	Yes	Yes	Yes	Yes
Other Controls	Yes	Yes	Yes	Yes
Observations	62782	62826	61611	12645

*Notes:* Estimations in this table account for all sample respondents including those without a job (earnings). Columns (1) to (3) report CRE estimation results with education level, perceived health, and emotional well-being disorder as outcome variables, respectively. Column (4) presents an OLS estimation of financial literacy as an outcome as data on financial literacy become available in wave 5. Other control variables used in the estimations are age, age squared, gender, marital status, and race. Robust standard errors are given in parentheses.**p < 0.01.

∗ p < 0.05.

∗∗∗ p < 0.001.

## 6 Conclusion

Previous literature on early-life shocks and late-life outcomes concentrates on relating covariate shocks such as rainfall variability, price volatility, war, and famine to health and education-related outcomes that include health status, education attainment, psychological or mental well-being, and cognitive ability. Rigorous studies exploring the effect of early life idiosyncratic shocks on labour market outcomes are rare, particularly in a developing country context. In this paper, we estimate the impact of early life idiosyncratic shocks on labour market outcomes. This paper extends the literature in two ways. First, idiosyncratic shocks which are specific to households are considered the main independent variables. Second, labour market outcomes are taken as outcome variables.

The key idiosyncratic shocks we consider are the death of a biological father and of a biological mother before the age of five. The main labour market outcome variable considered is monthly wage earnings, while unemployment is also used as an alternative indicator. The estimations used five waves of panel data from the National Income Dynamics Study (NIDS) (2008–2017) with a total observation of close to 63,000. We found that the mean difference in monthly wage earnings between those who lost their parents in childhood and those who did not is statistically significant. Consistently, while there is no statistically significant difference in the average wage earnings over time for those who experienced the shocks, there is statistically significant growth for those who did not experience the shocks.

Alternative estimations reveal that both the death of a biological father and the death of a biological mother in early childhood adversely affect wage earnings during adulthood, though the magnitude of the effect of the death of a mother is consistently higher than the death of a father. Results from heterogeneity analysis show that Black South Africans who lost their father in childhood earn higher wages than the other race categories that experienced the same shock. Associating the shock variables with unemployment indicates that the death of a mother is negatively related to both the probability of being unemployed and the probability of working which implies a positive association with the economically inactive category. However, the death of a father is negatively associated with the probability of working but positively associated with the probability of being unemployed. The main mechanisms through which the death of a mother affects wage earnings are education level, perceived health, cognitive ability, and higher probability of being employed in an elementary occupation. The adverse effect of the death of a father is mainly channelled through lowering education levels.

### 6.1 Policy implications

The results of this study clearly demonstrate the need for strengthening and aligning child support programmes to meet their intended goals. In South Africa, there is a child support programme, the foster child grant, that targets children who have lost both of their parents. This programme needs to target children who have lost either of their parents, particularly those who have lost their mother and are financially needy. The programme should also go beyond the financial grant to monitor its implementation in helping children in terms of their human capital formation. Psychological support for children should also be considered. The estimation results also imply that informal insurance, which is expected to minimize the impact of idiosyncratic shocks, does not function well to solve the problem. Therefore, it is also worth considering making households and the communities aware of the issue so that informal social protection plays a part in reducing the adverse consequences for children.

### 6.2 Limitations and further study directions

In analyzing the effect of early-life parental death on labour market outcomes, this study is limited to exploring and explaining associations rather than drawing causal interpretations. Causal interpretation necessitates a clear identification, where parental death should be either exogenous or at least be instrumented by exogenous variables. In the heterogeneity analysis, black South Africans who lost their biological father early in life are found to earn higher wages than the other race groups who have experienced the same shock. The study is constrained in its ability to investigate the reasons why. Therefore, future studies are likely to fill these gaps.

## Data availability

The raw data is publicly available at the Datafirst's website. Data and codes to replicate all the results will be available on request.

## CRediT authorship contribution statement

**Gidisa Lachisa Tato:** Writing – review & editing, Writing – original draft, Methodology, Formal analysis, Data curation, Conceptualization. **Assefa Admassie:** Conceptualization, Writing – original draft, Writing – review & editing, Supervision.

## Declaration of competing interest

The authors declare that they have no known competing financial interests or personal relationships that could have appeared to influence the work reported in this paper.

## Figures and Tables

**Table A.1:** Earning Variation by Education Categories

	(1)	(2)	(3)
	Mean	Std. dev.	Freq.
No formal education	1392.06	1475.48	1225
Primary education	1647.50	2107.00	2659
Secondary education	3075.31	5470.48	15708
Post-secondary education	9133.07	12678.75	4880
Total	4043.90	7654.60	24472

*Notes:* Column (1) shows the monthly wage earnings of the pooled data by education category. Column (2) presents the standard deviation of monthly earnings by the respective education categories, followed by Column (3) depicting the number of observations in each educational attainment category.

**Table A.2:** Mean Difference Tests by Shock Types and Age Groups

	(1)	(2)	(3)
	Age15-29	Age30-44	Age45+
Monthly Wage Earnings (in log) (Father Deceased)	0.073	0.143^∗∗^	0.404^∗∗∗^
	(1.16)	(2.72)	(6.45)
Monthly Wage Earnings (in log) (Mother Deceased)	0.291^∗^	0.233^∗^	0.503^∗∗∗^
	(2.40)	(2.30)	(4.86)
Observations	28981	17319	16615

*Notes:* The table provides mean difference tests for monthly wage-earning differences by age cohort. Column (1) is the result for the first working age group (age 15 to 29), while Columns (2) and (3) report mean difference tests for age groups 30–44 years, and 45 and older, respectively. t statistics are given in parentheses *p < 0.05, **p < 0.01, ***p < 0.001.

**Table A.3:** Mechanisms - Employed Sample and Parental Death before Age of 15

	(1)	(2)	(3)	(4)	(5)
	Education	PHealth	Emowellbeing	OccuElemd	FinLitw5
Death of a Father	−0.172^∗^	−0.027	0.128	0.100	0.025
	(0.087)	(0.020)	(0.079)	(0.059)	(0.037)
Death of a Mother	−0.658^∗∗∗^	−0.063^∗^	0.219	0.229^∗^	−0.180^∗∗∗^
	(0.125)	(0.029)	(0.124)	(0.090)	(0.054)
Time Fixed Effect	Yes	Yes	Yes	Yes	No
Municipality Fixed Effect	Yes	Yes	Yes	Yes	Yes
Other Controls	Yes	Yes	Yes	Yes	Yes
Observations	24282	24295	23764	23500	5764

*Notes:* Columns (1) to (3) report CRE estimation results with education level, perceived health and emotional well-being disorder as outcome variables, respectively. Column (4) shows the panel Probit estimation using an elementary occupation dummy as a dependent variable. Column (5) presents the OLS estimation of financial literacy as an outcome as financial literacy data becomes available in wave 5. Other control variables used in the estimations are age, age squared, gender, marital status, and race. Robust standard errors are given in parentheses. *p < 0.05, **p < 0.01, ***p < 0.001.
